# Association Between Census Tract-Level Poverty and Non-White Race with Location of Coal Ash Disposal Pits in the United States

**DOI:** 10.3390/ijerph22030408

**Published:** 2025-03-11

**Authors:** Emily A. Shingara, Caroline Weinberg, Tara P. McAlexander

**Affiliations:** 1 Department of Epidemiology and Biostatistics, Dornsife School of Public Health, Drexel University, Philadelphia, PA 19104, USA; tpm58@drexel.edu; 2 Earthjustice Clean Energy Program, Philadelphia, PA 19103, USA; cweinberg@earthjustice.org; 3 Urban Health Collaborative, Dornsife School of Public Health, Drexel University, Philadelphia, PA 19104, USA

**Keywords:** environmental health, coal ash, coal-fired power plants, socioeconomic status, environmental justice

## Abstract

Coal ash is a byproduct of coal-fired power plants, and the management and disposal of coal ash in coal ash pits is an environmental health concern. Evidence suggests that socioeconomically disadvantaged communities are more likely to bear the burden of these environmental hazards. However, limited studies have investigated the relationship between socioeconomic status and residential proximity to coal ash pits. We examined associations between census tract poverty and non-white race with the likelihood of having coal ash pits within the census tract. We obtained coal ash pit location and census tract-level data (2017–2021) of the percentage of the population living at or below the federal poverty level and the percentage of the population’s non-white race for 82,805 census tracts in the contiguous United States. We implemented multivariable logistic regression models to examine associations between non-white race, poverty, and the likelihood of having one or more coal ash pits in a census tract. Secondary analyses among tracts with at least one coal ash pit evaluated the associations between poverty, non-white race, and the likelihood of having multiple coal ash pits. Models additionally adjusted for census tract region. Census tracts in the highest quartile of poverty were over two times as likely to have one or more coal ash pits (OR = 2.23, 95% CI: 1.52, 3.25). Tracts in the highest quartile of the non-white population had 90% lower odds of having one or more coal ash pits as compared to the lowest quartile (OR = 0.10, 95% CI: 0.06, 0.17). Census tracts with higher levels of poverty were more likely to have one or more coal ash pits, and census tracts with higher non-white populations were less likely to have one or more coal ash pits, suggesting that these associations are complex and indicate an environmental justice issue.

## 1. Introduction

Coal ash is a waste mixture of highly concentrated carcinogens, neurotoxins, and other dangerous pollutants, including arsenic, boron, cadmium, chromium, cobalt, lead, lithium, mercury, radium, and thallium [1,2]. Coal ash, also known as coal combustion residuals (CCR), may appear in several forms, including fly ash, bottom ash, boiler slag, and flue gas desulfurization (FGD) sludge [1]. When these chemicals are inhaled or ingested, some can bioaccumulate in the fat, bones, and brains of the organisms that consume them [1]. The hazardous chemicals in coal ash can harm nearly every major organ in the body, causing cancer, kidney disease, heart and lung disease(s), nervous system damage, neurodevelopment difficulty, reproductive damage, and other suspected health risks [2,3].

Annually, the United States generates over 110 million tons of coal ash, largely produced by industrial coal-burning activities [2,3]. Coal-fired power plants and other industrial activities that produce coal ash have historically stored coal ash waste in unlined landfills and ash ponds, where a lack of barriers and regulations has allowed the coal ash to leak and consequently contaminate drinking water sources, surface water, and other environmental media [2]. In fact, analysis of industry-reported groundwater data reveals that over 90% of coal-fired power plants are polluting groundwater with one or more contaminants above health standards [2]. Additionally, fugitive fly ash from coal ash disposal facilities may contribute to ambient air pollution, which is particularly concerning for proximal residential communities [4]. While there are no federal fugitive dust policies, some states and localities have implemented their own regulations to address fugitive fly ash, such as stricter air quality standards in California and Colorado. These measures vary widely and often lack consistent enforcement, often depending on state or local political priorities and resources. Despite coal ash containing a hazardous concentration of pollutants, it has been disposed of almost entirely in landfills and surface impoundments located near residential communities without adequate regulations or safeguards to protect public health from being exposed [1]. Previous studies have demonstrated that coal ash pit proximity has been linked to adverse respiratory outcomes and poor school performance among children [4,5].

The United States Environmental Protection Agency (EPA) established a Final Rule on coal ash disposal practices in 2015, which consequently left over half of all coal ash unregulated in the United States [6]. The EPA finalized a new rule on coal ash disposal practices in April 2024, which will newly regulate hundreds of coal ash pits that had ceased receiving waste prior to the 2015 Rule. Many of these pits are located at the same power plants as pits regulated under the 2015 Rule, while others are located at power plant sites that were previously completely exempted from the rule. While this new rule is going into effect and its actual enforcement is still undetermined, research investigating sociodemographic factors related to the distribution of coal ash pits across the United States remains important to understand populations at the greatest risk of health impacts due to coal ash pits and their related exposures.

There is evidence that suggests that there are socioeconomic and racial disparities in health outcomes related to environmental pollution, specifically coal ash, which implicates environmental justice concerns [3,7,8,9]. The U.S. Commission on Civil Rights investigated the issue of coal ash and residential proximity in 2016. However, there are limited ecological level studies that investigate the relationship between socioeconomic status and racial identity with residential proximity to coal ash disposal pits.

To address this gap, we expand upon the findings reported by the U.S. Commission on Civil Rights since 2016 and data that has been made available since then through an empirical hypothesis-driven analysis to examine whether coal ash pits are more likely to be in communities that are traditionally socioeconomically disadvantaged, as indicated by higher levels of poverty and non-white populations. A secondary aim of this analysis was to evaluate whether, among census tracts with coal ash pits, poverty and non-white race were associated with the likelihood of having multiple coal ash pits compared to only one coal ash pit within the tract. We hypothesized that census tracts with a higher percentage of the population living at or below the federal poverty level and census tracts with a higher percentage of non-white population would have a greater prevalence of coal ash pits compared to census tracts that do not have a high percentage of the population living in poverty or a high percentage of non-white population.

## 2. Methods

This was an ecological study that utilized data from the United States Census American Community Survey (ACS) (five-year estimate data, 2017–2021) and data from Earthjustice, which provides locations of 746 coal ash pits throughout the United States (2015–2022). It should be noted that the Earthjustice database includes only those facilities that are federally mandated to report coal ash disposal data under the 2015 Rule. It does not include disposal pits that will be newly regulated under the 2024 Rule and, thus, may not be considered a comprehensive representation of all coal ash pits in the United States. Data from the American Community Survey was accessed through the Drexel University Urban Health Collaborative data repository.

Earthjustice is a nonprofit environmental law organization known for its transparency and history of legal victories that have led to cleaner air, the banning of harmful chemicals in food and homes, and various positive health outcomes for communities across the United States. This history of legal advocacy reinforces the credibility of their data, which has been used in numerous legal and policy initiatives aimed at improving environmental health.

### 2.1. Coal Ash Pits Locations Data

The Earthjustice database included information for 746 coal ash disposal pits across the United States that are federally regulated under the 2015 Rule. The coal ash pits included in this database are a mixture of active and inactive sites. As mentioned above, the 2015 Rule exempted many coal ash pits, so they are not represented in this database. The states that are not included because they did not have any coal ash pits regulated under the 2015 Rule are California, Connecticut, the District of Columbia, Hawaii, Idaho, Rhode Island, and Vermont. In our initial analysis, we include all states in the contiguous U.S., consistent with the availability of ACS data compiled into a geodatabase from the UHC. In the analysis of our second aim, we only include census tracts that have at least one coal ash pit.

These data sets were used to evaluate the association between socioeconomically disadvantaged communities, explicitly considering populations living at or below the federal poverty level and non-white populations, and the siting of coal ash pits. We summarized the number of coal ash pits throughout the United States at the census tract level. Our original dataset included 85,395 census tracts. Following a complete case analysis approach, we excluded census tracts that had missing data for our variables of interest (n = 1071) and census tracts in states from the Earthjustice dataset that were not in the contiguous United States (n = 1519). Our final analytic sample excluded the states of Alaska, Hawaii, and Puerto Rico and included 82,805 census tracts.

### 2.2. Measures

#### 2.2.1. Exposure Variables

The two primary exposure variables in this study were the percent of the population living at or below the federal poverty level (%poverty) and the percent of the population’s race (%race) in the census tract, which was used to represent socioeconomic disadvantage. We used both continuous and quartile measures of %poverty and %race in our analyses.

The percent of poverty in the dataset captures the percent of persons below the poverty level at all ages. In the ACS, %race was categorized by: Hispanic, non-Hispanic white, non-Hispanic black, non-Hispanic Asian, non-Hispanic other races, and two or more non-Hispanic races. It should be noted that communities with coal mining and related coal activities have historically been white and low-income [10]. However, we approached this analysis from a disparity perspective and assessed whether non-white populations were disproportionately burdened. For analysis purposes, in our adjusted models, we used %non-white in a census tract to account for racial/ethnic minorities, including those identified as Hispanic. Using %non-white theoretically reflects the percentage of racial/ethnic minority population and adds to 100% when combined with %white/non-Hispanic for purposes of a cumulative race variable.

We acknowledge the limitations and problematic nature of utilizing the term “non-white” in our research. While the term is employed for methodological reasons, we recognize that it creates an oversimplified racial binary that does not fully capture the diversity of racial and ethnic identities. We recognize that framing racial groups in contrast to whiteness can unintentionally reinforce a sense of “otherness” and marginalization. Despite these limitations, we use this term with an awareness of its implications and with the intent to critically examine racial disparities while remaining mindful of the complexities of racial and ethnic identities.

#### 2.2.2. Outcome

The presence of a coal ash pit in a census tract was the primary outcome of the study. For the analysis, we created a dichotomous outcome variable that indicates if there was one coal ash pit sited in the census tract or if there was more than one coal ash pit sited in the census tract. In aim 1, the outcome was having one coal ash pit sited in the census tract compared to those with no coal ash pits. In aim 2, the outcome was having more than one coal ash pit sited in the census tract compared to those with only one.

#### 2.2.3. Covariates

Covariates that may have confounded or influenced the relationship between socioeconomic disadvantage and coal ash pits were identified a priori to the analysis based on literature and directed acyclic graphs. The identified predictor was census region (Northeast, Midwest, South, West), as it was expected that region may relate to the racial and/or socioeconomic composition of a population of people in a census tract. By controlling for region, we aimed to isolate the effects of race and socioeconomic status on coal ash pit sitings, ensuring that observed outcomes are not reflections of broader regional trends. The measure of association between the covariate and the relationship of interest was assessed before and after adjustment.

### 2.3. Statistical Analysis

All statistical analyses were completed using SAS 9.4. We examined region, race, and poverty variables at the census tract level, stratified by the outcome of interest, using unadjusted descriptive statistics (frequencies/percentages, means/standard deviations). For our first aim analyses, we examined the odds of a census tract having one or more coal ash pits vs. none using multivariable adjusted logistic regression. In the first model of our primary analysis, we did not adjust for any additional variables and calculated the individual crude odds of %poverty and %non-white population with having one or more coal ash pits vs. none. In our second model, we adjusted for our a priori confounder of region. In our third and final model, we adjusted for region as well as a mutual adjustment for poverty and race, and we examined %poverty and %non-white population with the odds of having one or more coal ash pits vs. none. We used the same multivariable logistic regression and progressive adjustment models for our second aim analysis to examine %poverty and %non-white population with the odds of having more than one coal ash pit vs. only one coal ash pit per tract.

## 3. Results

Demographic information regarding census tract level characteristics in the 2017–2021 ACS used in the analysis can be found in Table 1. Of the 82,805 census tracts, 82,519 (99.6%) tracts did not have any coal ash pits, 286 (0.4%) had at least one coal ash pit, and 180 (0.2%) had more than one coal ash pit.

**Figure 1:**
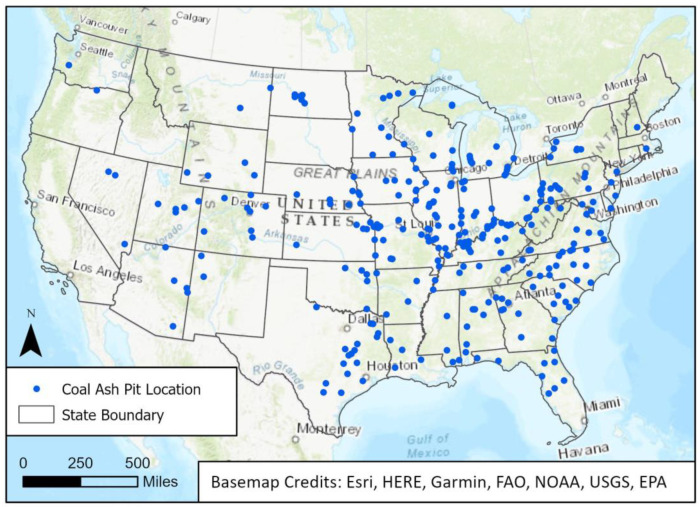
Geographic distribution of coal ash pits within the contiguous U.S.

The geographic distribution of coal ash pits within the contiguous U.S. can be seen in Figure 1, which reflects the data in the Earthjustice coal ash database. Regions with the most coal ash pits were the Midwest (n = 18,392) and South (n = 31,927). The Midwest region represented 39.9% (n = 114) of tracts with any coal ash pit and 42.8% (n = 77) of tracts with more than one coal ash pit (Table 1). Of the tracts with any coal ash pit in the Midwest, nearly half of them had one or more coal ash pits. The South represented 42.3% (n = 121) tracts with any coal ash pits, and 42.2% (n = 76) of tracts with more than one coal ash pit (Table 1). Although the West and Northeast regions had fewer coal ash pits overall compared to the Midwest and South, of the tracts with any coal ash pits in each region, roughly half of them had more than one coal ash pit.

The overall mean %white at the census tract level was 60.5%, and the overall mean %non-white was 39.5% (Table 1). The mean %white increased among tracts with any coal ash pit (78.6%) and among tracts with more than one coal ash pit (79.6%). The overall mean %poverty at the census tract level was 13.70%, which remained consistent among tracts without coal ash pits. The mean %poverty decreased among tracts with any coal ash pit (13.62%) and among tracts with more than one coal ash pit (12.80%).

In our primary and secondary analyses, %poverty was measured 0–100, and, therefore, our odds ratios were for a 1% difference in %poverty. Results of the final, fully adjusted model indicated a strong association between higher %poverty and the odds of a census tract having one or more coal ash pits vs. none (OR = 2.23, CI = 1.52, 3.25) (Model 3, Table 2). These results demonstrated that those in the highest (4th) quartile of %poverty had 2.23 times the odds of having one or more coal ash pits vs. none compared to the reference group (1st quartile of %poverty), indicating a strong association after adjusting for region and percent non-white, and followed the expected direction of our hypothesis: census tracts with higher levels of %poverty would have an increased likelihood of having a coal ash pit. The %poverty variable was also analyzed continuously as a sensitivity analysis, and the results were consistent with the primary analysis (Supplementary Materials). Specifically, in the final, fully adjusted model (Model 3, Table S1), the odds of having one or more coal ash pits vs. none were increased (OR = 7.18, CI = 2.59, 19.86).

In our primary and secondary analyses, %non-white was measured 0–100, and, therefore, our odds ratios were for a 1% difference in %non-white. Results of the final, fully adjusted model (Model 3, Table 2) indicated that those in the lowest (1st) quartile of %non-white had the highest odds of a census tract having one or more coal ash pits vs. none (Model 3, Table 2). Compared to quartile 1, tracts in quartile 2 had 53% lower odds of having at least one coal ash pit (OR = 0.47, 95% CI: 0.35, 0.63), 67% lower odds in quartile 3 (OR = 0.33, 95% CI: 0.24, 0.47), and 90% lower odds in quartile 4 (OR = 0.10, 95% CI: 0.06, 0.17). These results demonstrate that as the quartiles of %non-white increase, the odds of a census tract having one or more coal ash pits vs. none decrease. The direction of this association did not follow the expected direction of our hypothesis: census tracts with higher levels of %non-white population would have increased the likelihood of having a coal ash pit. The %non-white variable was also analyzed continuously as a sensitivity analysis, and the results were consistent with the primary analysis (Supplementary Materials). Specifically, in the final, fully adjusted model (Model 3, Table S1), the odds of having one or more coal ash pits vs. none were reduced (OR = 0.04, CI = 0.02, 0.07).

Results from our secondary analysis indicated that there was not a clear association between %poverty and likelihood of having multiple coal ash pits in a census tract, in contrast to findings from our primary analysis (Table 2). The final, fully adjusted model revealed that, as compared to the 1st quartile of %poverty (reference group), tracts in quartiles 2, 3, and 4 did not have any statistically significant associations between %poverty and having multiple coal ash pits in a census tract (Table 2). The results of the sensitivity analysis where we analyzed %poverty continuously were consistent with those of our secondary analyses assessing the association between %poverty and the odds of census tracts having more than one coal ash pit vs. only one (Model 3, Table S2).

Results from both our primary and secondary analyses of %non-white were similar, suggesting that, overall, across the contiguous United States, and then restricted to only tracts that had a coal ash pit, we observed the same associations. Results of the final, fully adjusted model (Model 3, Table 2) indicated that those in the lowest (1st) quartile of %non-white had the highest odds of a census tract having one or more coal ash pits vs. none (Model 3, Table 2). Compared to quartile 1, tracts in quartile 2 had 53% lower odds of having at least one coal ash pit (OR = 0.47, 95% CI: 0.35, 0.63), 67% lower odds in quartile 3 (OR = 0.33, 95% CI: 0.24, 0.47), and 90% lower odds in quartile 4 (OR = 0.10, 95% CI: 0.06, 0.17). These results demonstrate that as the quartiles of %non-white increase, the odds of a census tract having one or more coal ash pits vs. none decrease. The direction of this association did not follow the expected direction of our hypothesis: census tracts with higher levels of %non-white population would have an increased likelihood of having a coal ash pit. The results of the sensitivity analysis were consistent with those of our secondary analyses assessing the association between %non-white and the odds of a census tract having more than one coal ash pit vs. only one (Model 3, Table S2).

### Correlation of Variables

We performed a multicollinearity test between %poverty and %non-white. The Pearson correlation coefficient was 0.40 with a *p*-value of <0.0001. These results indicate a statistically significant (*p*-value < alpha = 0.5), positive relationship between %poverty and %non-white. However, 40% collinearity between these two variables did not suggest a need to separate them for analysis purposes and implies independent relationships between both variables and the outcome(s) of interest.

## 4. Discussion

In this study, we investigated the association between census tract-level poverty and non-white race with the location of coal ash disposal pits. Our work aimed to expand upon the findings reported by the U.S. Commission on Civil Rights in 2016, utilizing updated coal ash pit and census data to further explore the relationship between socioeconomic disadvantage and the siting of coal ash pits within census tracts. In our analyses, we measured socioeconomic disadvantage by combining census tract measures of %poverty and %non-white, which allowed us to better examine the ways in which these measures may intersect and affect health both exclusively and mutually.

Previous research by the U.S. Commission on Civil Rights has shown that of populations living within one mile of a coal ash disposal facility, over 16% of residents were part of a racial/ethnic minority group and over 13% were low-income (live below the Federal Poverty Level), which may be determined disproportionally high only when compared to national averages (24.8% minority and 11.3% living below Federal Poverty Level, nationally) [9]. The EPA and U.S. Commission on Civil Rights have not confirmed that coal ash facilities are disproportionately located in the vicinity of communities that are low-income and/or have a higher proportion of racial/ethnic minorities, although the results of our study may contribute to the body of literature which describe a relationship between poverty within a census tract and increased coal related activities [9]. Although we intended to perform this study from a disparities perspective to investigate our hypotheses, our findings are consistent with previous research, which do not indicate disproportionate sitings in racial/ethnic minority census tracts. Previous analyses have found that over 70% of coal plants were surrounded by populations whose racial/ethnic minority representation fell below that of the state population, further suggesting that coal-fired utilities are located in zip codes where the population is disproportionately white than where it is disproportionately racial/ethnic minority [9].

We recognize that communities with coal mining and related coal activities have largely been historically white and low-income [10]. Although %non-white population was not an indicator at the census tract level for coal ash pits in this study, %poverty is a metric of social disadvantage that may still capture non-white affected communities, as this study was performed at the census tract level and cannot capture individual burdens within them. Communities are often segregated by socioeconomic status and race, suggesting that they commonly share area-level characteristics of socioeconomic disadvantage. It should be emphasized that the related health disparities from environmental hazards are traditionally disproportionately experienced among non-white populations [9,10]. Our findings do not discredit that there may be individual facilities that have intentionally chosen a site for a coal ash landfill or pond because of its proximity to socially disadvantaged communities. In fact, other analyses have shown that those coal ash facilities where the surrounding communities exceed state averages for certain social disadvantages also tend to be the most densely populated communities and, thus, account for a large percentage of the overall impacted population [11]. Additionally, the utilization of census tracts in this study may not describe the individual characteristics and impacts of residents closest to those disposal pits, only the proportion of those among the entire census tracts. Finally, it is worth noting that about 60% of coal-fired electricity generation in the US is located in rural areas and that rural areas in the US are well-documented to be, on average, almost two times as white as urban areas [12,13]. Thus, the %non-white analysis in this study may largely reflect these rural vs. urban patterns.

As of 25 April 2024, the EPA announced changes to the CCR regulations for inactive coal ash surface impoundments at inactive facilities and for CCR management units, referring to the “legacy disposal pits” which had previously been omitted from the Final Coal Ash Rule in 2015 if they had stopped receiving coal ash before the Rule went into effect [6,14]. This updated rule will force power plants to monitor and clean up previously exempted coal ash disposal pits that have been leaking toxic pollution into the groundwater, a critical revision to a rule that had otherwise left over half of all coal ash exempt from federal clean-up requirements [6]. Although this new regulation aims to improve current and future conditions in which coal ash disposal is regulated for the protection of both environmental and human health, power plants have been improperly disposing of coal ash for decades, and the siting of these coal ash facilities may still be inflicting disproportionate impacts on proximal socioeconomically disadvantaged communities.

The siting of coal ash pits is linked to broader historical patterns of energy production and consumption in the United States. Coal-fired power plants are increasingly being phased out of use in favor of natural gas and renewable energy sources. This shift aligns with national and global efforts to reduce contributions to greenhouse gas emissions and mitigate the effects of climate change. However, the coal ash disposal pits that remain are not only a persistent environmental concern for surrounding communities, but they may also be particularly vulnerable to the effects of climate change. With the increase in intense storms, flooding, sea-level rise, and extreme weather events related to climate change, there is an increase in the potential for contaminant leaching and fugitive dust dispersion from these coal ash pits. These risks underscore the need for proactive environmental management and policy interventions. Given these dynamics, this study’s investigation of coal ash pit sitings and their relationship to race and socioeconomic status is particularly timely, as it highlights how the burdens of past energy infrastructure decisions continue to shape environmental inequities.

The strengths of our study are that it is more up-to-date than other studies, including the years 2017–2021, and contributes to a growing body of literature investigating the association between socioeconomic disadvantage and the siting of coal ash pits [2,3,4,5,9]. Our results are consistent with previous studies and reinforce the strength of those findings. Lastly, our study utilized nationally representative data and included a large sample size. However, the nature of an area-level study inherently has limitations, particularly at the census tract level, which may not capture individual-level burdens from environmental hazards (i.e., coal ash pits). In this instance, a census tract may not indicate a high proportion of non-white population, but the individuals in that census tract who identify as non-white may either live in closer proximity to a coal ash disposal site or experience an increased burden, which we cannot describe in the results of this study. The missing census tract data from this study, including states excluded from having no coal ash pits regulated under the 2015 Rule, census tracts with missing data for variables of interest in the study, and those excluded from the contiguous United States, although we expect their effects to be marginal, are a limitation to this study in our capacity to entirely describe the relationship between socioeconomic disadvantage and a coal ash pit siting across the United States.

## 5. Conclusions

Overall, we found that census tracts with higher levels of poverty had increased odds of having a coal ash pit, and reduced associations were found between %non-white population in a census tract and having a coal ash pit. Due to the nature of coal-related activities in the United States and their historical relationship with low-income white communities, these results should not overshadow the implications that coal ash disposal regulations have on socioeconomically disadvantaged communities, particularly those in racial/ethnic minority groups. As the U.S. transitions from coal-fired power, the legacy of coal ash pits remains a significant challenge, exacerbated by shifting political priorities that create regulatory uncertainty.

Stronger federal and state regulations on fugitive coal ash, stricter enforcement, and increased oversight are essential to reducing exposure risks. Climate adaptation measures must also ensure that coal ash sites are resilient to extreme weather. Additionally, empowering affected communities through public engagement and transparent policymaking is crucial to addressing systemic inequities.

This study highlights the need for targeted policy interventions that safeguard vulnerable communities and uphold environmental protections amid political and energy landscapes. Future research is needed to enhance the strength of evidence-based policies regulating the disposal of coal ash. Additional studies are needed to further examine coal ash exposure risks in the context of climate change and shifting regulatory frameworks to prevent ongoing environmental injustices. This issue remains pertinent to future public health work, as coal-fired power plants and other industrial activities that produce coal ash may continue improperly storing coal ash and actively compromising public health.

## Figures and Tables

**Table 1:** Descriptive statistics of contiguous U.S. census tracts.

	Total Tracts	Tracts Without Coal Ash Pits	Tracts with Any Number of Coal Ash Pits	Tracts with More than One Coal Ash Pit
Overall (N)	82,805	82,519	286	180
Variables at the census tract level				
Region	N (%)	N (%)	N (%)	N (%)
Northeast	14,493 (17.50)	14,473 (17.54)	20 (6.99)	10 (5.56)
Midwest	18,392 (22.21)	18,278 (22.15)	114 (39.86)	77 (42.78)
South	31,927 (38.56)	31,806 (38.54)	121 (42.31)	76 (42.22)
West	17,993 (21.73)	17,962 (21.77)	31 (10.84)	17 (9.44)
Race	Mean (SD)	Mean (SD)	Mean (SD)	Mean (SD)
%White	60.5 (29.63)	60.4 (29.64)	78.6 (21.23)	79.6 (19.62)
%Non-white, other race(s) *	39.5 (29.63)	39.6 (29.64)	21.4 (21.23)	20.4 (19.62)
Poverty				
%Poverty **	13.70 (11.35)	13.70 (11.35)	13.62 (9.10)	12.80 (8.06)

* %non-white, other race(s) = Hispanic, non-Hispanic Black, non-Hispanic Asian, non-Hispanic other race(s), and non-Hispanic multiple race(s). ** %poverty = percent population living at or below federal poverty level.

**Table 2:** Estimated likelihood of having at least one coal ash pit vs. none and more than one coal ash pit vs. only one coal ash pit.

	Model 1: Unadjusted	Model 2: Adjusted for Region	Model 3: Model 2 + Mutual Adjustment for Poverty and Race
	OR (95% CI)	OR (95% CI)	OR (95% CI)
Estimated likelihood of a census tract having one or more coal ash pits vs. none			
%Poverty *			
1st quartile (reference) (0, 0.06)	-	-	-
2nd quartile (>0.06, 0.11)	1.52 (1.07, 2.18)	1.48 (1.04, 2.12)	1.53 (1.07, 2.19)
3rd quartile (>0.11, 0.19)	1.86 (1.32, 2.63)	1.80 (1.28, 2.55)	2.11 (1.49, 3.0)
4th quartile (>0.19, 1.0)	1.34 (0.92, 1.94)	1.25 (0.87, 1.81)	2.23 (1.52, 3.25)
%Non-white **			
1st quartile (reference) (0, 0.14)	-	-	-
2nd quartile (>0.14, 0.32)	0.46 (0.34, 0.61)	0.47 (0.35, 0.63)	0.47 (0.35, 0.63)
3rd quartile (>0.32, 0.62)	0.35 (0.25, 0.48)	0.37 (0.26, 0.51)	0.33 (0.24, 0.47)
4th quartile (>0.62, 1.0)	0.11 (0.07, 0.19)	0.12 (0.07, 0.21)	0.10 (0.06, 0.17)
Estimated likelihood of a census tract having more than one coal ash pit vs. only one coal ash pit per tract			
%Poverty *			
1st quartile (reference) (0, 0.06)	-	-	-
2nd quartile (>0.06, 0.11)	0.96 (0.46, 2.03)	0.97 (0.46, 2.06)	0.95 (0.45, 2.02)
3rd quartile (>0.11, 0.19)	1.24 (0.60, 2.56)	1.28 (0.62, 2.68)	1.24 (0.59, 2.60)
4th quartile (>0.19, 1.0)	0.65 (0.31, 1.38)	0.66 (0.31, 1.42)	0.66 (0.30, 1.43)
%Non-white **			
1st quartile (reference) (0, 0.14)	-	-	-
2nd quartile (>0.14, 0.32)	0.46 (0.34, 0.61)	0.47 (0.35, 0.63)	0.47 (0.35, 0.63)
3rd quartile (>0.32, 0.62)	0.35 (0.25, 0.48)	0.37 (0.26, 0.51)	0.33 (0.24, 0.47)
4th quartile (>0.62, 1.0)	0.11 (0.07, 0.19)	0.12 (0.07, 0.21)	0.10 (0.06, 0.17)

* %poverty = percent population living at or below federal poverty level. ** %non-white, other race(s) = Hispanic, non-Hispanic Black, non-Hispanic Asian, non-Hispanic other race(s), and non-Hispanic multiple race(s).

## Data Availability

The ACS 2017–2021 5-year data is a publicly available source [15]. The Earthjustice coal ash database is also a publicly available source [16].

## Supplementary Materials

The following supporting information can be downloaded at: https://www.mdpi.com/article/10.3390/ijerph22030408/s1, Table S1: Estimated likelihood of a census tract having 1 or more coal ash pits vs. none, continuous measurement of variables for sensitivity analyses; Table S2: Estimated likelihood of a census tract having 1 or more coal ash pits vs. only 1 coal ash pit per tract, continuous measurement of variables for sensitivity analyses.

## Abbreviations

Coal combustion residuals (CCRs); Flue gas desulfurization (FGD).
