# Supplementary material for: Association Between Census Tract-Level Poverty and Non-White Race with Location of Coal Ash Disposal Pits in the United States
Source: Int J Environ Res Public Health. 2025 Mar 11;22(3):408. doi: 10.3390/ijerph22030408 (PMC11942556; doi:10.3390/ijerph22030408)
Supplement: Supplementary file 1 [file ijerph-22-00408-s001.zip › ijerph-3439485-supplementary.pdf]

Supplemental Material

Association Between Census Tract Level Poverty and Non-white Race with Location of Coal Ash Disposal Pits in the United States

Supplemental Table S1: Estimated likelihood of a census tract having 1 or more coal ash pits vs. none, continuous measurement of variables for sensitivity analyses

Table S1. Estimated likelihood of a census tract having 1 or more coal ash pits vs. none

	**Model 1: Unadjusted**	**Model 2: Adjusted for Region**	**Model 3: Model 2+ mutual adjustment for poverty and race**
	**OR (95% CI)**	**OR (95% CI)**	**OR (95% CI)**
**%Poverty***	0.94 (0.33, 2.62)	0.95 (0.34, 2.66)	7.18 (2.59,19.86)
**%Non-white***	0.06 (0.03, 0.10)	0.05 (0.03, 0.09)	0.04 (0.02, 0.07)

* %poverty= percent population living at or below federal poverty level
** %non-white, other race(s)= Hispanic, non-Hispanic Black, non-Hispanic Asian, non-Hispanic other race(s), & non-Hispanic multiple race(s)

Supplemental Table S2: Estimated likelihood of a census tract having 1 or more coal ash pits vs. only 1 coal ash pit per tract, continuous measurement of variables for sensitivity analyses

Table S2. Estimated likelihood of a census tract having 1 or more coal ash pits vs. only 1

	**Model 1: Unadjusted**	**Model 2: Adjusted for Region**	**Model 3: Model 2+ mutual adjustment for poverty and race**
	**OR (95% CI)**	**OR (95% CI)**	**OR (95% CI)**
**%Poverty***	0.07 (0.01, 1.00)	0.07 (0.01, 1.05)	0.08 (0.01, 1.48)
**%Non-white***	0.56 (0.18-1.70)	0.56 (0.17-1.86)	0.88 (0.24-3.25)

* %poverty= percent population living at or below federal poverty level
** %non-white, other race(s)= Hispanic, non-Hispanic Black, non-Hispanic Asian, non-Hispanic other race(s), & non-Hispanic multiple race(s)
